# Supplementary material for: Performance of Three Tests for SARS-CoV-2 on a University Campus Estimated Jointly with Bayesian Latent Class Modeling
Source: Microbiol Spectr. 2022 Jan 19;10(1):e01220-21. doi: 10.1128/spectrum.01220-21 (PMC8768831; doi:10.1128/spectrum.01220-21)
Supplement: SUPPLEMENTAL FILE 1 — Supplemental material. Download SPECTRUM01220-21_Supp_1_seq12.pdf, PDF file, 0.7 MB [file spectrum01220-21_supp_1_seq12.pdf]

Supplemental Material For

Performance of three tests for SARS-CoV-2 on a university campus estimated jointly with Bayesian latent class modeling

T. Alex Perkins[1*], Melissa Stephens[2], Wendy Alvarez Barrios[3], Sean Cavany[1], Liz Rulli[3], Michael E. Pfrender[1*]

[1]Department of Biological Sciences, University of Notre Dame, Notre Dame, IN, USA
[2]Genomics and Bioinformatics Core Facility, University of Notre Dame, Notre Dame, IN, USA
[3]Notre Dame Research, University of Notre Dame, Notre Dame, IN, USA
*Authors for correspondence: taperkins@nd.edu, mpfrende@nd.edu

SUPPLEMENTAL TEXT

Prior distributions

Regarding the prior probabilities of the parameters, Pr($\boldsymbol{\theta}$), we assumed a uniform prior for all sensitivity and specificity parameters. We explored uniform priors for the prevalence parameters but found that such a choice could result in very high estimates of prevalence and very low estimates of sensitivity and specificity, which seemed implausible based on other studies (1, 2). To reflect our assumption that the tests are likely more accurate than not and that infection prevalence is likely to be relatively low, we adopted beta distributed priors with shape parameters 1 and 99 for individuals recruited for surveillance testing. These parameters correspond to a distribution with a mean infection prevalence of 0.01 skewed towards lower values, which is generally consistent with model-based estimates of infection prevalence for St. Joseph County, Indiana (2). For individuals tested for non-surveillance purposes, we adopted beta distributed priors with shape parameters 1 and 9, which correspond to a distribution with a mean infection prevalence of 0.10 skewed towards lower values. Values in that range are generally consistent with test positivity for the state of Indiana (3).

Markov chain Monte Carlo algorithm

To avoid the challenges associated with calculating Pr($\boldsymbol{n}$) directly, we approximated the posterior distribution of $\boldsymbol{\theta}$ using Markov chain Monte Carlo. Specifically, we used the Metropolis-Hastings algorithm as implemented with default settings in the BayesianTools (4) package in R (5). We ran a total of 100,000 iterations across nine chains, applying a burnin at 10,000 iterations for each and thinning every 100 samples. We assessed convergence through visual inspection of traceplots and calculation of Gelman-Rubin statistics (Fig. S3). We assessed parameter non-identifiability through pairwise correlation plots (Fig. S4).

Estimation of time-varying prevalence

To obtain an estimate of time-varying prevalence, *Prev*(*t*), among the campus population, we first estimated daily incidence of infection, I_S(*t*), from the time series of symptomatic case notifications, as described in (6). To do this, we deconvolved the symptomatic case notifications with the incubation period distribution and the delay from symptom onset to testing. The incubation period was modeled as a log-normal distribution with parameters μ = 1. 621 and σ = 0.418 (7), and the delay from symptom onset to testing as a Poisson distribution with a mean of two days. We used the backprojNP function in the R surveillance package (version 1.18.0) for the deconvolution (8). We then estimated the total number of infections by date of infection, $I(t) = I_S(t)\ /\ 0.57,$ by assuming that 57% of infections were symptomatic (9) and that all symptomatic infections were ultimately tested under the intense on-campus testing environment. Finally, we estimated *Prev*(*t*) as

$$Prev(t) = \sum_{\tau=-\infty}^{t} I(\tau) Se(t-\tau),$$

where *Se*(*t*) is an estimate of sensitivity by day of infection by Grassly et al. (10). Although it would have been more ideal to make use of data that speaks directly to asymptomatic

infections, the use of surveillance testing was too inconsistent over the course of the semester to inform estimates of time-varying patterns of asymptomatic infection incidence. However, one independent data stream that supports our extrapolation of time-varying patterns of symptomatic infections comes from SARS-CoV-2 RNA concentrations from wastewater samples, which were collected consistently throughout the semester and display similar trends as symptomatic infection incidence (6).

Model validation

Overall, 98% of the 800 simulated parameter values (8 parameters x 100 simulated data sets) fell within their respective 95% credible intervals. For all individual parameters, 95% or more of simulated values fell within their 95% credible intervals. These results suggest that our posterior estimates provide an appropriate description of uncertainty about the true values of the parameters that we sought to estimate. In addition, median values from the posterior distribution were well correlated with simulated values. Pearson correlations ranged from 0.40 for saliva sensitivity to 0.76 for saliva specificity (Fig. S2). Within the range of simulated parameter values we considered, these results suggest that the inference method produces median estimates in the right general direction but that true parameters may lie elsewhere within the credible interval.

SUPPLEMENTAL TABLES

Table S1. Test positivity stratified by test type (columns) and reason participants were tested (rows).

	Commercial	Saliva	Antigen
**Surveillance positivity**	1.2% (10/802)	2.5% (20/805)	0% (0/3)
**Non-surveillance positivity**	6.5% (2/31)	14.6% (6/41)	14.7% (5/34)

Table S2. Pairwise probabilities that one type of test (row) is more sensitive than another (column).

**Sensitivity**	Commercial	Saliva	Antigen
Commercial	-	0.31	0.71
Saliva	0.69	-	0.88
Antigen	0.29	0.12	-

Table S3. Pairwise probabilities that one type of test (row) is more specific than another (column).

**Specificity**	Commercial	Saliva	Antigen
Commercial	-	0.86	0.92
Saliva	0.14	-	0.81
Antigen	0.08	0.19	-

SUPPLEMENTAL FIGURES

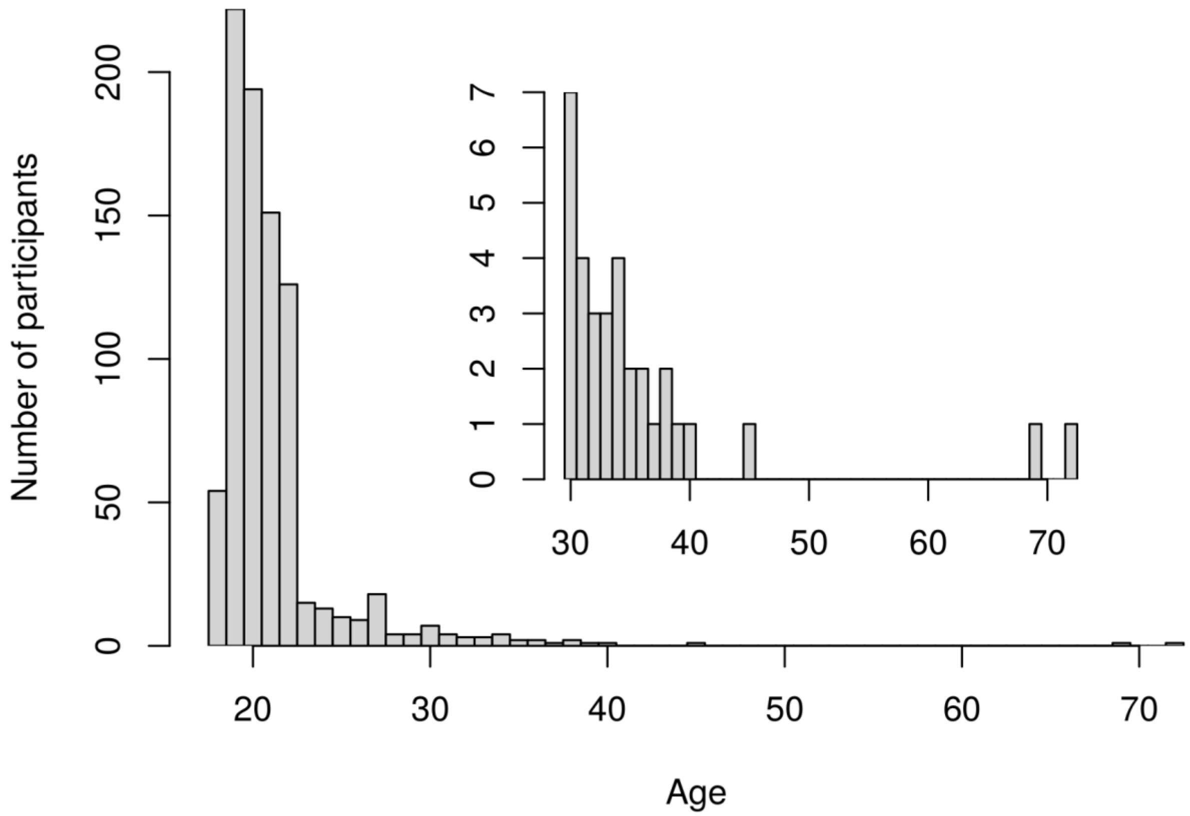

Figure S1. Age distribution of participants. The main panel shows the overall age distribution of study participants, while the inset panel shows the age distribution for individuals 30 and older.

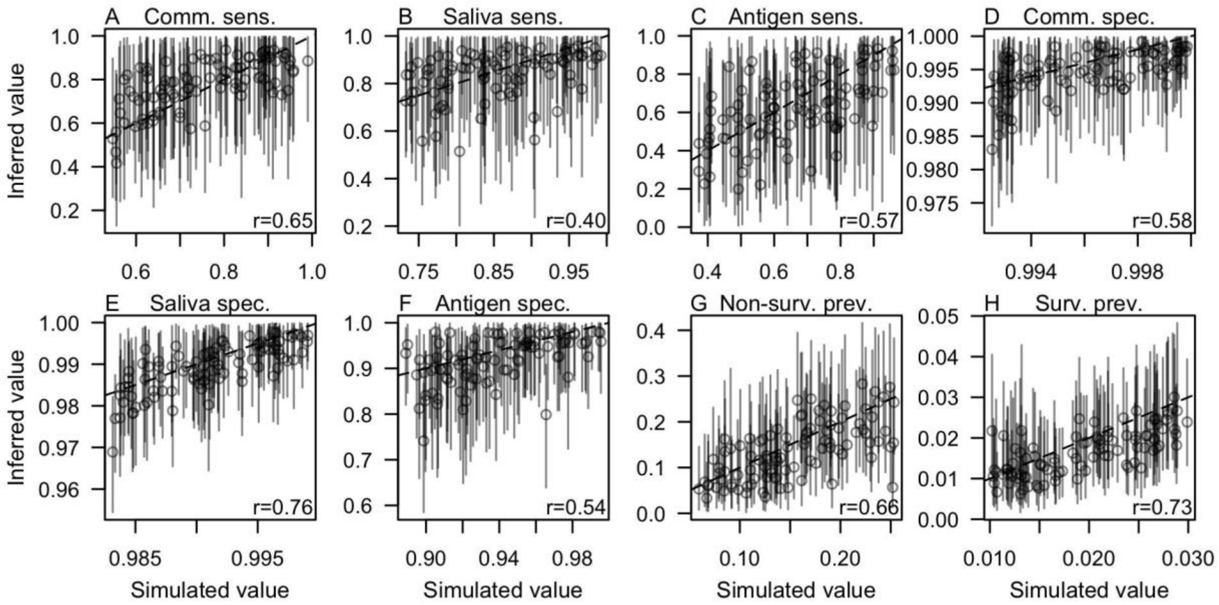

Figure S2. Validation on simulated data. For each of 100 parameter sets drawn uniformly and independently from the 95% credible interval of each parameter (x-axis), we simulated testing outcomes for each individual from each category (i.e., reason for testing, which tests were applied) in our empirical data set. For each of those simulated data sets, we obtained posterior estimates of the eight parameters, the median and 95% credible intervals of which are displayed here (y-axis). The dashed line shows a one-to-one relationship, and the Pearson correlation between simulated and median inferred values for each parameter is displayed in each panel.

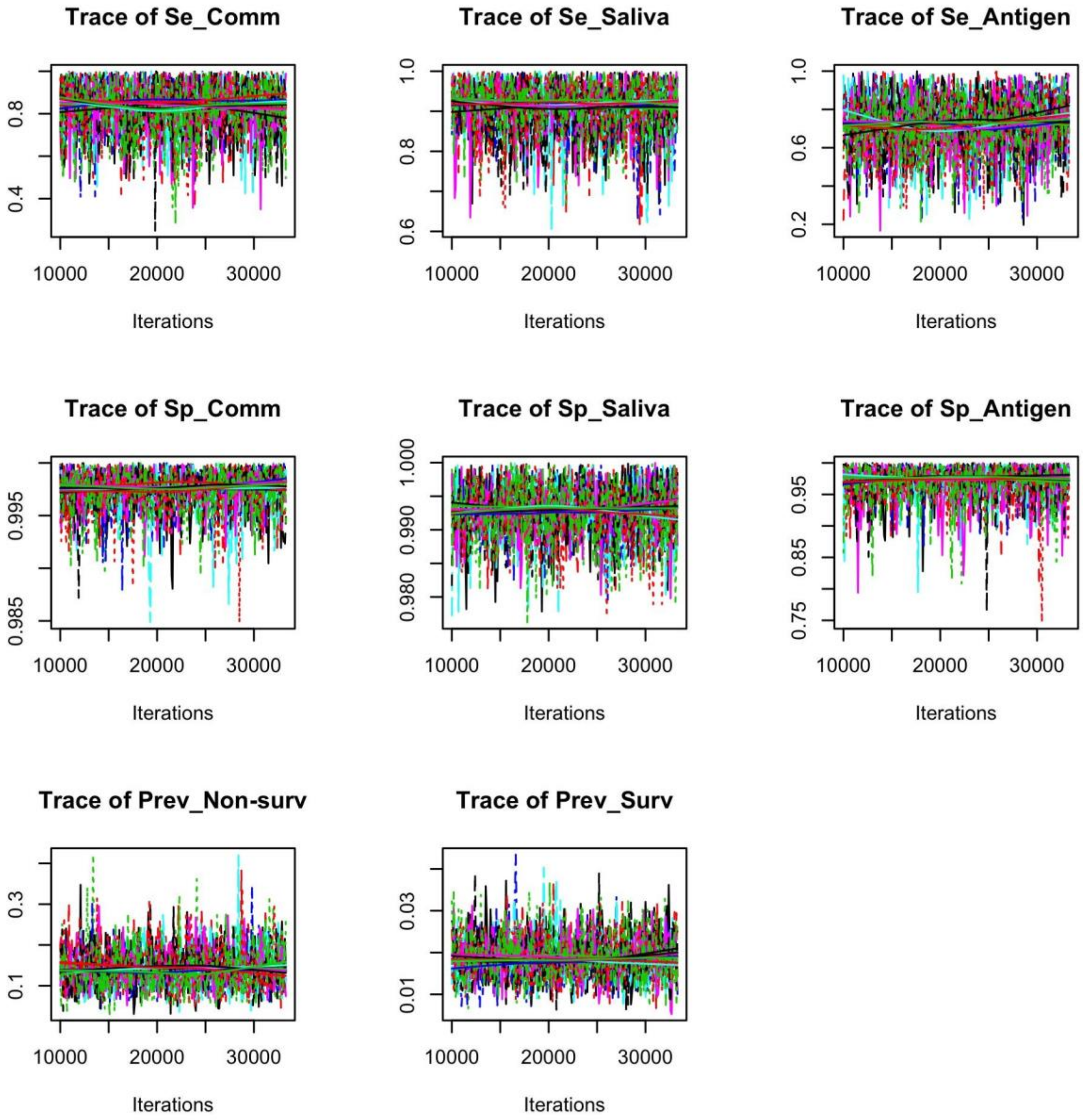

Figure S3. Trace plots of the eight parameters. Traces of the model parameters over the course of the MCMC chains reveal no visual indication of autocorrelation. This interpretation is supported by potential scale reduction factors being at or below 1.01 for all parameters (both for point estimates and upper confidence intervals) and the multivariate potential scale reduction factor being 1.01, as assessed by the gelmanDiagnostics function from the BayesianTools package in R.

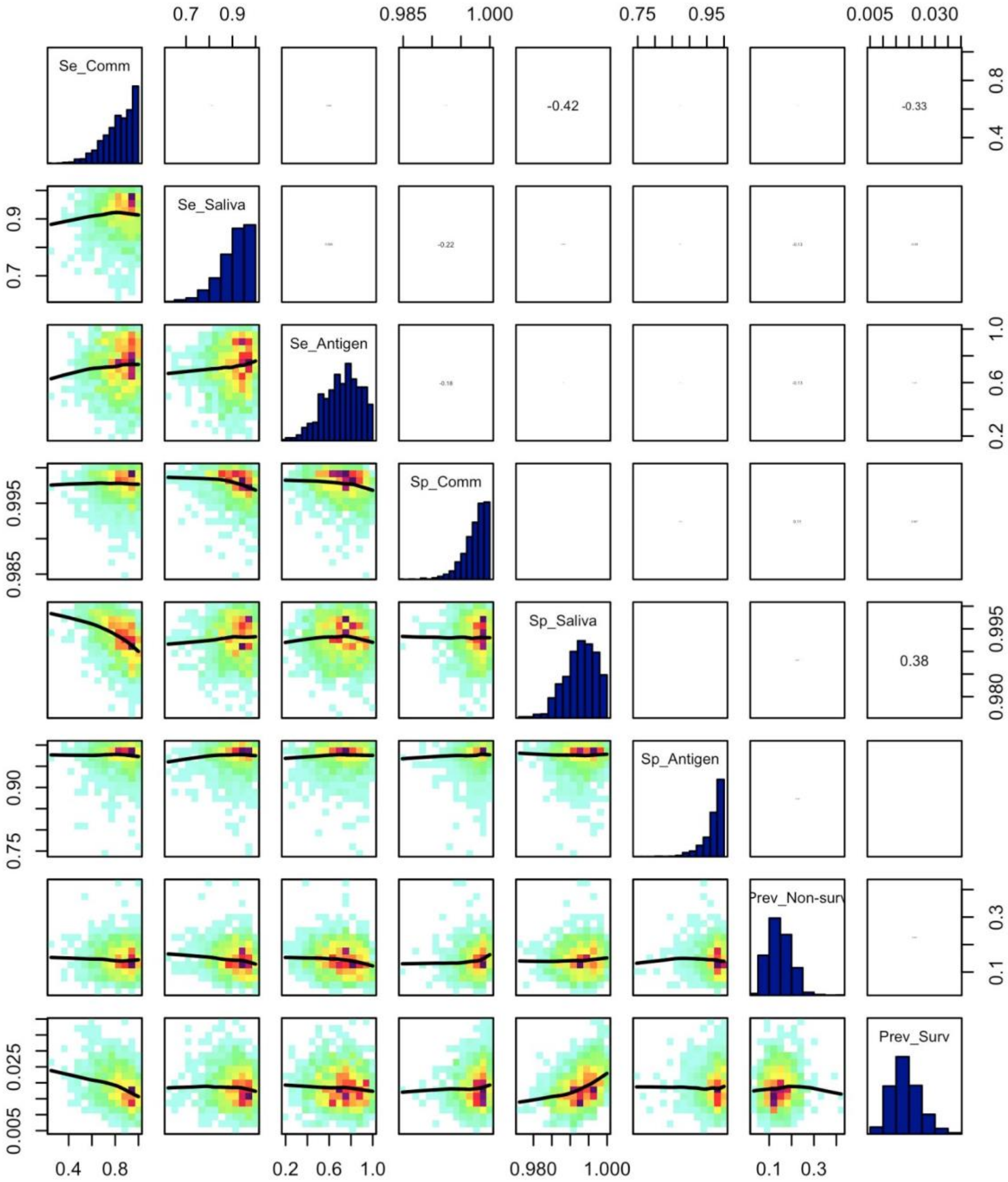

Figure S4. Correlation plot for the eight parameters. The most prominent correlations occurred among Se_{Comm}, Sp_{Saliva}, and $Prev_{Surv}$. This indicates some discrepancy between commercial and saliva tests about samples collected through surveillance. Specifically, this pattern is consistent with samples with a positive result under the saliva test but a negative result under the commercial test. The existence of these correlations indicates some degree of difficulty in determining the true latent state for samples for which that inconsistency occurs. This figure was made with the correlationPlot function from the BayesianTools library in R.

SUPPLEMENTAL REFERENCES

1. Butler-Laporte G, Lawandi A, Schiller I, Yao M, Dendukuri N, McDonald EG, Lee TC. 2021. Comparison of Saliva and Nasopharyngeal Swab Nucleic Acid Amplification Testing for Detection of SARS-CoV-2: A Systematic Review and Meta-analysis. JAMA Intern Med 181:353–360.

2. Pei S, Yamana TK, Kandula S, Galanti M, Shaman J. 2021. Burden and characteristics of COVID-19 in the United States during 2020. Nature 598:338-341.

3. 2021. ISDH - Novel Coronavirus: Indiana COVID-19 Dashboard and Map.

4. Hartig F, Minunno F, Paul S. 2018. BayesianTools: General-Purpose MCMC and SMC Samplers and Tools for Bayesian Statistics, R package version 0.1. 3.

5. R Core Team. 2018. R: A Language and Environment for Statistical Computing. R Foundation for Statistical Computing, Vienna, Austria.

6. Cavany S, Bivins A, Wu Z, North D, Bibby K, Perkins TA. 2021. Inferring SARS-CoV-2 RNA shedding into wastewater relative to time of infection. medRxiv.

7. Lauer SA, Grantz KH, Bi Q, Jones FK, Zheng Q, Meredith HR, Azman AS, Reich NG, Lessler J. 2020. The Incubation Period of Coronavirus Disease 2019 (COVID-19) From Publicly Reported Confirmed Cases: Estimation and Application. Ann Intern Med 172:577–582.

8. Meyer S, Held L, Höhle M. 2017. Spatio-Temporal Analysis of Epidemic Phenomena Using the R Package surveillance. Journal of Statistical Software.

9. Kasper MR, Geibe JR, Sears CL, Riegodedios AJ, Luse T, Von Thun AM, McGinnis MB, Olson N, Houskamp D, Fenequito R, Burgess TH, Armstrong AW, DeLong G, Hawkins RJ,

Gillingham BL. 2020. An Outbreak of Covid-19 on an Aircraft Carrier. N Engl J Med 383:2417–2426.

10. Grassly NC, Pons-Salort M, Parker EPK, White PJ, Ferguson NM, Imperial College COVID-19 Response Team. 2020. Comparison of molecular testing strategies for COVID-19 control: a mathematical modelling study. Lancet Infect Dis 20:1381–1389.
